# Estimating Loss of Brucella Abortus Antibodies from Age-Specific Serological Data In Elk

**DOI:** 10.1007/s10393-017-1235-z

**Published:** 2017-05-15

**Authors:** J. A. Benavides, D. Caillaud, B. M. Scurlock, E. J. Maichak, W. H. Edwards, P. C. Cross

**Affiliations:** 1 Department of Ecology, Montana State University, 310 Lewis Hall, Bozeman, MT 59717 USA; 2 Institute of Biodiversity Animal Health and Comparative Medicine, University of Glasgow, Glasgow, G12 8QQ UK; 3 The Dian Fossey Gorilla Fund International, Atlanta, GA USA; 4 Wyoming Game and Fish Department, Pinedale, WY 82941 USA; 5 Wyoming Game and Fish Department, Laramie, WY 82071 USA; 6 U.S. Geological Survey, Northern Rocky Mountain Science Center, 2327 University Way Suite 2, Bozeman, MT 59715 USA; 7 Department of Anthropology, The University of California, Davis, Davis, CA 95616 USA

**Keywords:** Antibody loss, Approximate Bayesian computation, Brucellosis, Greater Yellowstone Ecosystem, Basic reproduction number, Serology

## Abstract

Serological data are one of the primary sources of information for disease monitoring in wildlife. However, the duration of the seropositive status of exposed individuals is almost always unknown for many free-ranging host species. Directly estimating rates of antibody loss typically requires difficult longitudinal sampling of individuals following seroconversion. Instead, we propose a Bayesian statistical approach linking age and serological data to a mechanistic epidemiological model to infer brucellosis infection, the probability of antibody loss, and recovery rates of elk (*Cervus canadensis*) in the Greater Yellowstone Ecosystem. We found that seroprevalence declined above the age of ten, with no evidence of disease-induced mortality. The probability of antibody loss was estimated to be 0.70 per year after a five-year period of seropositivity and the basic reproduction number for brucellosis to 2.13. Our results suggest that individuals are unlikely to become re-infected because models with this mechanism were unable to reproduce a significant decline in seroprevalence in older individuals. This study highlights the possible implications of antibody loss, which could bias our estimation of critical epidemiological parameters for wildlife disease management based on serological data.

**Electronic supplementary material:**

The online version of this article (doi:10.1007/s10393-017-1235-z) contains supplementary material, which is available to authorized users.

## Introduction

Many disease monitoring programs in wildlife rely on serology to infer disease prevalence (Gilbert et al. 2013). A positive serological response indicates previous exposure to a pathogen because specific antibody titers can remain at a detectable concentration long after an initial infection (Fredriksen et al. 1999). If antibodies are long-lived, then serological data can be used to estimate the force of infection as well as the vaccination coverage that may be required for eradication (Farrington et al. 2001; Hens et al. 2010). However, the probability of antibody loss following infection is generally unknown for most wildlife systems (Gilbert et al. 2013). This can potentially result in biased estimates of epidemiological parameters such as the *R*_0_, which is the expected number of infections caused by a typical infectious individual in a completely susceptible population (Anderson et al. 1992). In humans, antibodies to common viral and vaccine antigens persist for extended periods of time (Amanna et al. 2007). By contrast, antibodies to other pathogens, particularly bacteria, decline several months or years after the first infection [e.g, pertussis (Edwards 2005), cholera (Clements et al. 1982), chlamydia (Gijsen et al. 2002)].

Estimating the probability of antibody loss, referred here as the annual probability of a previously infected seropositive individual becoming seronegative, typically requires following individuals for several years post-infection (Clements et al. 1982; Fredriksen et al. 1999). This also requires ensuring that the individual does not get re-exposed. Given logistical constraints, this is typically not feasible for most wildlife systems. Alternatively, the probability of antibody loss can be estimated using novel statistical methods consisting in fitting mechanistic simulation models to observed patterns (Toni et al. 2009). In this study, we use a similar Bayesian modeling approach to Toni et al. (2009) to fit different disease scenarios to age-specific seroprevalence data in order to estimate the annual probability of antibody loss.

The purpose of this study is to estimate the probability of antibody loss and to investigate the duration of post-infection immunity to brucellosis in elk (*Cervus canadensis*) using empirical age–seroprevalence data. Brucellosis, a bacterial disease, is the most common zoonotic infection worldwide (Pappas et al. 2006) and prevention in humans requires a good understanding of its circulation in animal reservoirs (Seleem et al. 2010). In the USA, brucellosis caused by *Brucella abortus* remains in the free-ranging elk and bison (*Bison bison*) populations around the Greater Yellowstone Ecosystem (GYE). Brucellosis continues to threaten the livestock economy in the area when transmission from wildlife to cattle occurs (Cross et al. 2010). Disease transmission likely occurs from February to June, when susceptible individuals become infected through contact with an infected aborted fetus, placenta or fluids (Cheville et al. 1998). Campaigns to limit brucellosis in elk include vaccination with strain 19 on Wyoming’s 21 feedgrounds and the National Elk Refuge since 1985 (Herriges et al. 1992; Scurlock and Edwards 2010), whereby over 90% of juvenile elk (less than 2 years old) are vaccinated with a biobullet. However, current vaccines induce poor protection from brucellosis in elk compared to bison or cattle, because they do not induce robust and persistent immunological response in this species (Herriges et al. 1992; Olsen et al. 2006).

Previous studies have suggested that older bison could experience antibody loss (Joly and Messier 2004; Treanor et al. 2011). In addition, decreasing antibody titers have been observed for vaccination strains in elk (Olsen et al. 2006). However, no studies have attempted to quantify antibody loss in free-ranging elk. Furthermore, antibody loss could result in at least two very distinct epidemiological scenarios. Antibody loss can be accompanied by a loss of adaptive humoral immunity to a particular pathogen after it has been removed by the immune response, allowing the individual to become infected again when re-exposed to the same pathogen (Edwards 2005). Alternatively, antibody loss can be followed by an adaptive cell-mediated immune response conferring long-term immunity without involving antibodies (Takaki et al. 2000), which is a mechanism suggested for intracellular bacteria such as *Brucella* spp. (Yingst and Hoover 2003). Here, we intend to discriminate between the above two epidemiological scenarios confronting three different models of brucellosis dynamics: (1) a brucellosis epidemiological model without antibody loss, (2) a model with antibody loss and further re-exposure and (3) a model with antibody loss and lifelong immunity. Our models assumed no vertical transmission from an infected mother to its offspring [as previously described for elk brucellosis (Thorne et al. 1978)] nor disease recrudescence (shown in humans (Pellicer et al. 1988) but not described for elk). Given the vaccination campaigns to control brucellosis in elk on Wyoming feedgrounds (Maichak et al. 2017), we also focused on estimating how antibody loss can affect the calculation of the basic reproduction number *R*_0_. From *R*_0_, we estimated the associated vaccination coverage needed to eradicate the disease, if a more effective vaccine were to become available.

## Methods and Models

### Empirical Data

Three datasets were used in this study for different purposes: (1) The first dataset was used to reconstruct the age–seroprevalence curve, estimate the effect of age on seroprevalence and estimate antibody loss using the approximate Bayesian computation-sequential Monte Carlo (ABC-SMC) model fit to age–seroprevalence data, (2) the second dataset included individuals tested over multiple years to estimate antibody loss independently of the ABC-SMC, and (3) the third dataset was used to estimate brucellosis disease-induced mortality from collared animals. The first dataset included 478 serologic assays for exposure to brucellosis of elk females captured from the Grey’s River feedground near Alpine, Wyoming, USA. Details on blood sample collection, serological methods and the empirical data used in this study are provided in Sect. 1 of Supplementary Material. Grey’s River elk population sizes were extracted from annual reports of fixed-wing aerial trend counts (Wyoming Game and Fish Department 2007). In this feedground, vaccination with strain 19 has been implemented since 1985, reaching more than 95% coverage of all calves (Scurlock and Edwards 2010). The second dataset included 107 female elk that were tested during multiple years from different feedgrounds. This dataset included a total of 243 samples (with 25 antibody losses) collected from ten different Wyoming feedgrounds between 1995 and 2011. The third dataset included 258 collared female elk from 21 feedgrounds and other areas of Wyoming provided by Wyoming Game and Fish Department (WGFD) that were tagged from 2007 to 2012 and tested for brucellosis serological status when the collar was attached and removed. Collars were attached between January and March and deployed during one to three years.

### Statistical Analyses

The effect of age on seroprevalence was tested from the first dataset using a generalized linear model (GLM) with a binomial error and a logit link function using the software R v.3.2.0 (R Development Core Team 2014). This model also included the year of sampling as a categorical independent variable, which controlled for annual variation in brucellosis transmission. We also tested separately the effect of the elk population size per year using the above variables as controls. Since 22% of individuals were sampled more than once, results of this model were also confirmed with a generalized linear model choosing randomly only one sample per individual, which produced similar results.

### Antibody Loss Estimation From the Age–Seroprevalence Curve Using the ABC-SMC

#### Brucellosis Transmission Models

We compared the ability of three different epidemiological models to recreate the age–seroprevalence curve observed in our first dataset by developing three different stochastic discrete-time individual-based models representing the spread of brucellosis within females of an elk population: (1) a susceptible-infectious-recovered (SIR) model with no antibody loss, referred as the ‘No antibody loss’ model, (2) a SIRS model with antibody loss and loss of immunity, referred as the ‘Antibody loss and loss of immunity’ model and (3) a SIRN model with antibody loss and lifelong immunity, referred as the ‘Antibody loss and lifelong immunity’ model. Individuals in the *N* class recover from the disease, are no longer susceptible to reinfection, but are seronegative (Table 1). In these models, the recovered category represents individuals that are seropositive but are no longer infectious. We assumed that individuals in the *I* and *R* classes tested positive for antibodies, whereas individuals in the *S* and *N* classes tested negative. For the last two models, an additional version was also implemented including a multi-compartmental ‘box-car’ approach (Keeling and Rohani 2008) to create recovered periods (class *R*) that were roughly log-normally distributed (Lloyd 2001; Wearing et al. 2005). These models were referred to as ‘Slow antibody loss and loss of immunity’ and the ‘Slow antibody loss and lifelong immunity’ models. They illustrate scenarios where antibody loss occurs several years after recovering from the disease, because antibodies usually decrease for several years before reaching an undetectable level in humans (Farrell et al. 1975). To simplify the analyses, we did not account for year-to-year variation, but ran the model to a steady state. We modeled the probability of transmission to a given susceptible in year *t* as 1 − (1 − *β*)^*I*(*t*)^. Mortality rates and population size (*N* = 800 individuals) were constant in the model, because we do not expect demographic parameters to affect the shape of the age–seroprevalence curve and therefore the estimation of the probability of antibody loss. Additional details on all models are provided in Sect. 3 of Supplementary Material. *R*_0_ was approximated in each model to $R_{0} \approx \beta \times Z \times \frac{{\left( {1 - \left( {\gamma + \left( {1 - \gamma } \right) \times \bar{\mu }} \right)} \right)}}{{\left( {\gamma + \left( {1 - \gamma } \right) \times \bar{\mu }} \right)}}$ with *β* = transmission probability, *Z* = total population size*, γ* = *recovery probability* and $\bar{\mu }$ = average of the age-specific mortality probabilities weighted by the age distribution (details are given in Sect. 4 of Supplementary Material). The vaccination coverage needed to eradicate the disease was calculated as $1 - \frac{1}{{R_{0} }}$ (Dietz 1993).

**Table 1:** Simulated Scenarios of Within-Host Brucellosis and Prior Distribution of Parameters.

Simulated scenario	Model type	Model structure*	Parameters and prior distribution values
No antibody loss	SIR	${\text{s}}\mathop{\mathop{\longrightarrow}\limits}\limits^{\beta}{\text{l}}\mathop{\mathop{\longrightarrow}\limits}\limits^{\gamma}{\text{R}}$	β: Uniform[0;1] *γ*: Beta[5,6]
Antibody loss and loss of immunity	SIRS	${\text{S}}\mathop{\mathop{\longrightarrow}\limits}\limits^{\beta}{\text{l}}\mathop{\mathop{\longrightarrow}\limits}\limits^{\gamma}{\text{R}}\mathop{\longrightarrow}\limits^{\theta}{\text{S}}$	β: Uniform[0;1] *γ*: Beta[5,6] θ: Uniform[0;1]
Slow antibody loss and loss of immunity	SIRS with box-car waiting time on R	${\text{S}}\mathop{\mathop{\longrightarrow}\limits}\limits^{\beta}{\text{l}}\mathop{\mathop{\longrightarrow}\limits}\limits^{\gamma}{\text{R}}_{1} \mathop{\mathop{\longrightarrow}\limits}\limits^{\theta} \cdots \mathop{\mathop{\longrightarrow}\limits}\limits^{\theta}{\text{R}}_{5} \mathop{\mathop{\longrightarrow}\limits}\limits^{\theta}{\text{S}}$	
Antibody loss and lifelong immunity	SIRN	${\text{S}}\mathop{\mathop{\longrightarrow}\limits}\limits^{\beta}{\text{l}}\mathop{\mathop{\longrightarrow}\limits}\limits^{\gamma}{\text{R}}\mathop{\mathop{\longrightarrow}\limits}\limits^{\delta}{\text{N}}$	β: Uniform[0;1] *γ*: Beta[5,6] δ: Uniform[0;1]
Slow antibody loss and lifelong immunity	SIRN with box-car waiting time on R	${\text{S}}\mathop{\mathop{\longrightarrow}\limits}\limits^{\beta}{\text{l}}\mathop{\mathop{\longrightarrow}\limits}\limits^{\gamma}{\text{R}}_{1} \mathop{\mathop{\longrightarrow}\limits}\limits^{\delta} \cdots \mathop{\mathop{\longrightarrow}\limits}\limits^{\delta}{\text{R}}_{5} \mathop{\mathop{\longrightarrow}\limits}\limits^{\delta}{\text{N}}$	

* Individuals will transit from the *S* class to *I* with transmission probability *β*, from the *I* class to *R* with recovery probability *γ*, from the *R* class back to *S* with antibody loss probability *θ* and from the *R* class back to *N* with antibody loss probability *δ*. Mortality rate was set to *µ* = [0.3, 0.1, 0.5] for ages 1, 2–18 and 19+ , respectively, in all models

#### Parameter Estimation Using ABC-SMC

Traditionally, epidemiological parameters have been estimated from the age–seroprevalence curve using different versions of the catalytic model (Hens et al. 2010), allowing maximum-likelihood estimations. However, the catalytic model applies generally to age–seroprevalence curves increasing with age and is often constrained by assumptions such as long-lived antibodies (Caley and Hone 2002; Grenfell and Anderson 1985; Heisey et al. 2006; Hens et al. 2010). This approach is not suited to disentangle more complex scenarios of antibody loss such as the ones described above, where disease prevalence could also decay in older individuals. The difficulty of writing a closed-form likelihood function of the age–prevalence data given the parameters (especially for the model with slow antibody loss) complicates the use of standard MCMC techniques for model inference. Instead, we used an approximate Bayesian computation-sequential Monte Carlo (ABC-SMC) approach (Toni et al. 2009) to estimate epidemiological parameters from the age–prevalence curve obtained from the first dataset and to compare models of within-host brucellosis. The ABC method replaces the calculation of the likelihood with a comparison between the observed and simulated data using a ‘distance metric.’ Briefly, the distance metric used here was the sum of squared differences between the observed and simulated prevalence at each age category weighted by its sample size, given by ${\text{SS}} = \mathop \sum \nolimits_{i = 1}^{19} \left( {e_{i} - o_{i} } \right)^{2} \times A_{i}$ where *A*_*i*_ is the number of samples collected at age *i* and *e*_*i*_ and *o*_*i*_ are the simulated and observed prevalence at age *i*, respectively. Further details of this procedure are provided in Sect. 3 of Supplementary Material.

### Antibody Loss Estimation from Animals Sampled Multiple Years

Independently from the ABC-SMC, we used the second dataset including multiple year sampling to estimate the probability of antibody loss using a time-to-failure approach in a GLM. Details of this analysis are provided in Sect. 2 of Supplementary Material.

### Disease-Induced Mortality Analysis

Brucellosis is not known to cause acute mortality, but it is associated with lesions in the joints and arthritis, which may increase mortality rates of a prey species and is a potential alternative mechanism for a declining seroprevalence in older individuals (Thorne et al. 1978). Survival was estimated from the third dataset using both a GLM and a Cox proportional hazard (CPH) model approach (details on Sect. 5 of Supplementary Material). The same significant results were obtained using a CPH mixed effect model including individual ID as random effect using the coxme package in R.

## Results

**Figure 1:**
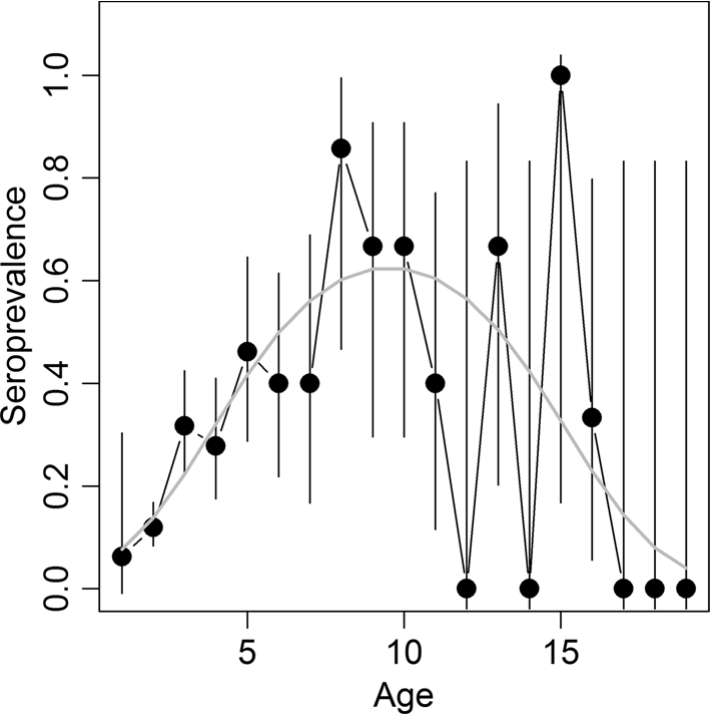
Empirical age–seroprevalence curve for female elk. The *gray line* represents the prediction based on the GLM model where seroprevalence is explained by age, age^2^ and year of collection. *Error bars* correspond to the 95% confidence interval. Sample sizes for age classes 1 to 19 years old were 16, 234, 82, 54, 26, 20, 10, 7, 6, 6, 5, 1, 3, 1, 1, 3, 1, 1, 1, respectively.

The total seroprevalence of brucellosis in elk females with known ages captured from the Grey’s River feedground was 23.8% (114/478), with an expected percentage of false positives being less than 8% based on a test specificity of 90% and a sensitivity of 100% (Gall et al. 2001). The age–seroprevalence curve of brucellosis estimated from the first dataset in female elk had a concave down shape, with a decrease in seroprevalence in females aged 10 and older (Figure 1). A model explaining serological status with a second-degree polynomial effect of age and sampling year (GLM with binomial error, age^2^ estimate ± SD: −11.21 ± 2.84, z-value _2456_ = −3.75, *P* < 0.001) fit the data better than a model including only age and sampling year as independent variables (log-likelihood test, *X* ^2^ = 19.52, d*f* = 1, *P* < 0.001). During the study period, Grey’s River feedground had an average ± SD elk population size of 792 ± 169, ranging from 491 to 1100 individuals. Seroprevalence was not affected by the elk population size (GLM including age, year of sampling and population size). A second-degree polynomial on age was significant in all combinations of model tested (including or not year of sampling and population size), and the shape of the curve remained unchanged when controlling for sampling year.

The probability of antibody loss estimated from the second dataset including females sampled multiple times was 0.07 year ^−1^ [95% CI 0.05–0.11]. Among 25 females who lost their antibody titers, 18 lost them within the first subsequent test. Considering that 8% of these 18 events could be attributed to false positives (see Sect. 2 of Supplementary Material), we reran this last analysis randomly excluding two samples (which encompassed the 8% false positives) and found no significant difference in the estimates.

We found no evidence for an influence of serological status on elk survival using a GLM to analyze the collar data of 258 female elk (mortality rate for seronegative individuals = 0.03 [95% CI 0.00–0.16], positive status odds ratio = 0.87 [95% CI 0.28–2.52]). Likewise, no influence of serological status was found using the CPH analysis (positive status odds ratio = 0.79 [95% CI 0.32–1.93]). Therefore, we did not include brucellosis-induced mortality in the simulation models. As an additional test to independently assess the potential role of disease-induced mortality, we estimated the age–seroprevalence curve using the upper end of the 95% confidence interval of the estimated disease-induced mortality from the above analyses (i.e., odds ratio = 2.52). This was achieved using the Heisey et al. (2006) formula, which is, to our knowledge, one of the only estimation methods of the age–seroprevalence curve with disease-induced mortality. We found that disease-induced mortality alone cannot generate a decrease in seroprevalence in older individuals (results shown in Sect. 5 of Supplementary Material).

**Figure 2:**
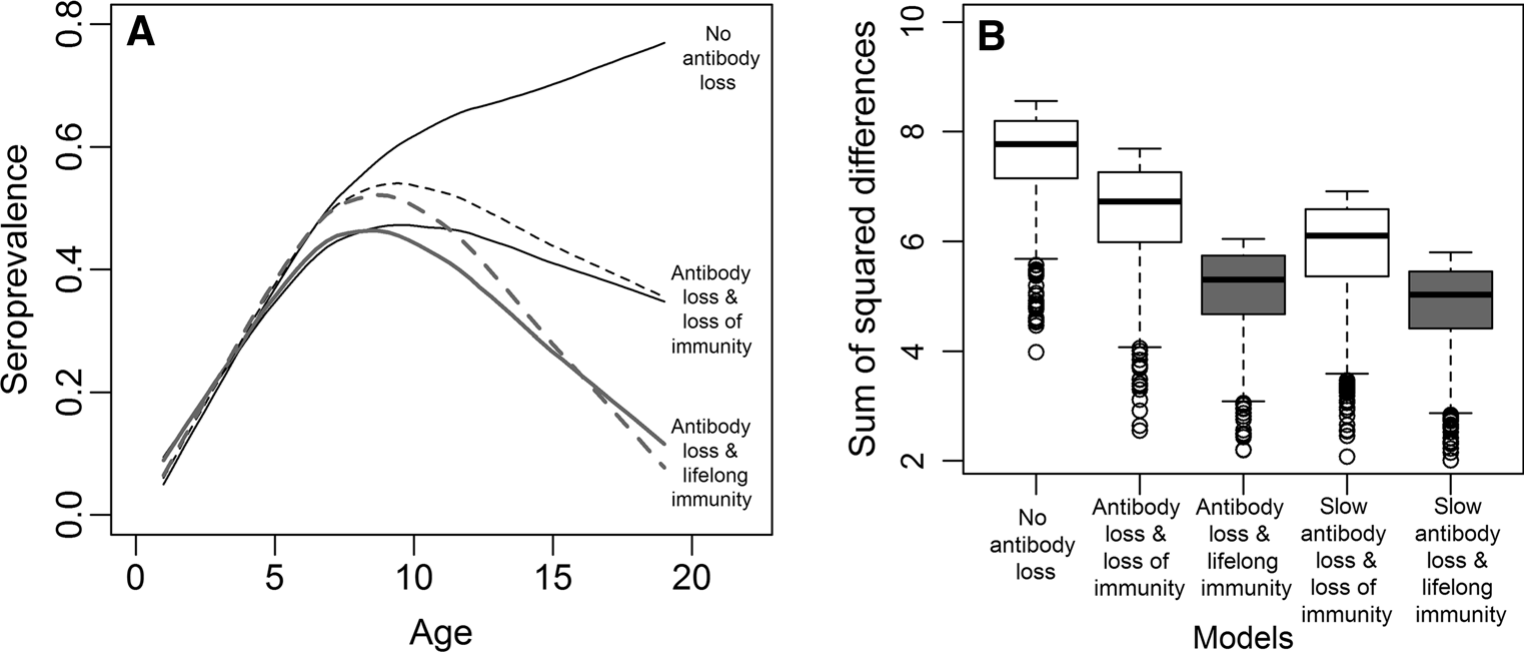
Predicted age–seroprevalence curve by different alternative models of within-host brucellosis dynamics. In (A), each curve is predicted by different models using the loess smooth function averaging the 1000 best simulations selected by the ABC-SMC procedure. *Dashed lines* represent models with a waiting time in the R class (i.e., ‘Slow Antibody loss models’). *Gray lines* represent the two models showing a significant decline in seroprevalence in older individuals, also shown in *gray-colored* boxplots on the plot (B) for comparison. In (B), the sum of squared differences between the empirical and the predicted data (SS metric) is summarized from the best 1000 simulations selected by the ABC-SMC. Boxplot shows the median, 25% quartiles and extreme values.

**Figure 3:**
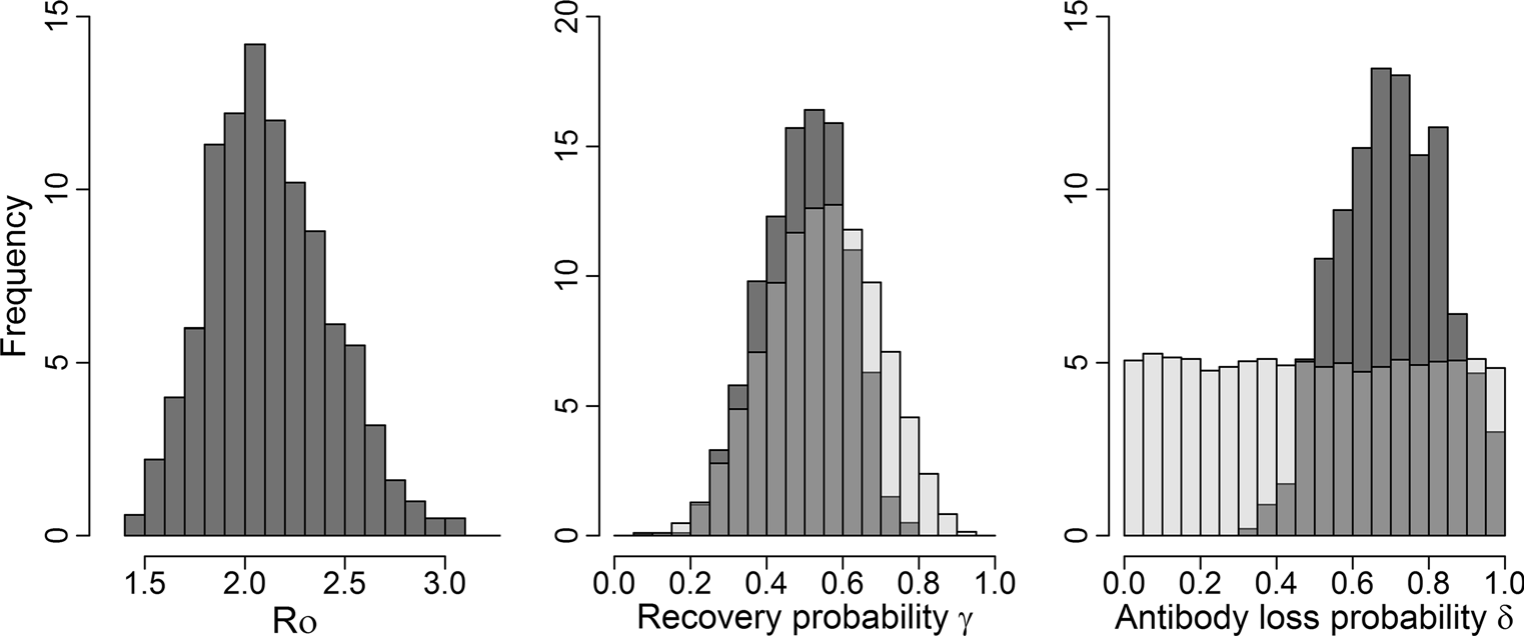
Posterior distribution for the SIRN-box model. Posterior (*dark gray bars*) and prior (*light gray bars*) distributions for each parameter (*R*_0_, recover probability *γ* and antibody loss probability δ) estimated using the ABC-SMC procedure are shown for the ‘Slow antibody loss and lifelong immunity’ model. A strong prior distribution was chosen for *γ* following Thorne et al. (1978). The prior distribution for the parameter *R*_0_ is not shown given its wide range (0–177, median = 14.35).

Only the SIRN models including lifelong immunity after antibody loss predicted a significant decline in the seroprevalence of older individuals as observed in the empirical data (Figure 2a). The SIR model generated a monotonic increase in seroprevalence with age, while models with antibody loss and loss of immunity predicted a minor decline in the seroprevalence of older individuals. This result was also reflected on the goodness-of-fit SS metric used in the ABC-SMC procedure, where the ‘Slow antibody loss and lifelong immunity’ model fit the empirical data the best (Figure 2b) and had an estimated annual probability of antibody loss around 0.70, with zero probability of being smaller than 0.3 (Figure 3). In different versions of this model, a five box-car model (mean ± SD of SS = 4.84 ± 0.76) fit the data marginally better than the three (mean ± SD of SS = 5.07 ± 0.77) or four (mean ± SD of SS 5.02 ± 0.75) box-car models. Posterior distributions for all models are provided in supplementary Table S1.

**Figure 4:**
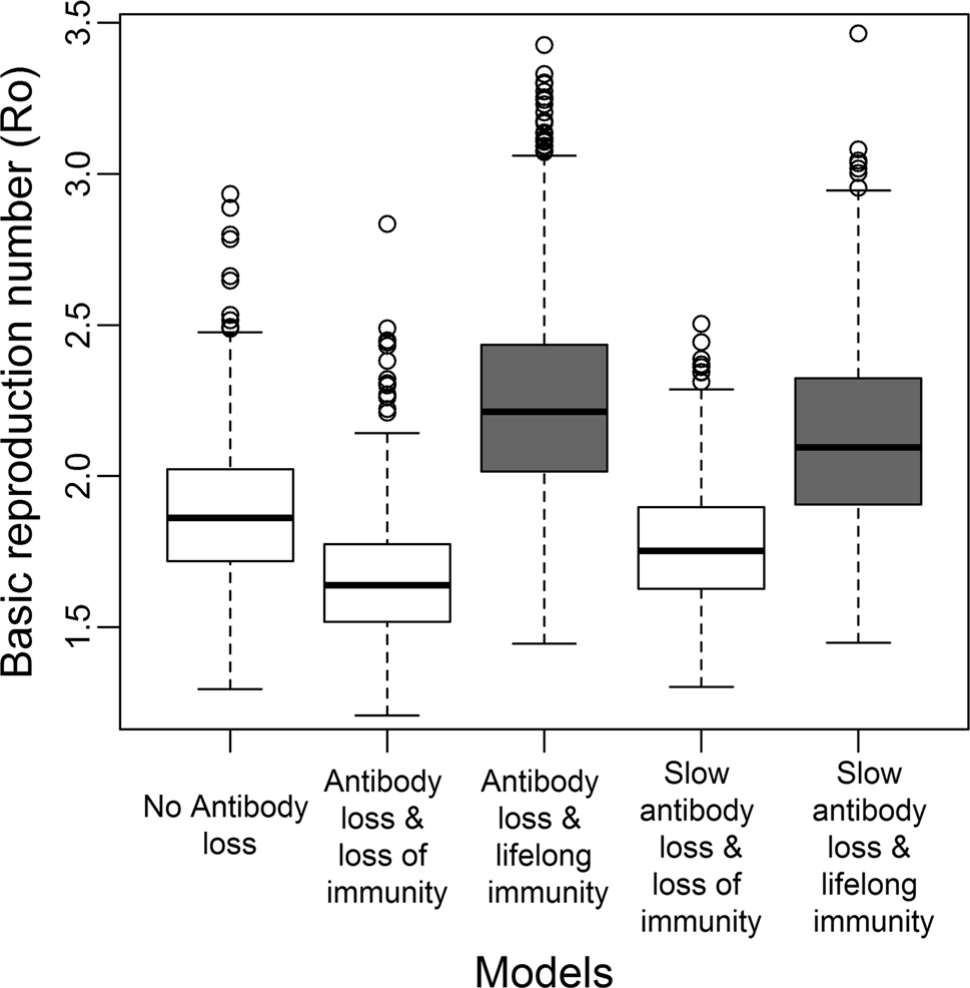
Comparison of *R*_0_ estimations in different models. The estimated *R*_0_ considering only females are infectious in the population is summarized from the best 1000 simulations selected by the ABC-SMC procedure for each model. Boxplots generated using R show the median, 25% quartiles and extreme values. For comparison with Figure 1, *gray-colored* boxplots indicate models with antibody loss and lifelong immunity. Details on the calculation of *R*_0_ are provided in Sect. 5 of Supplementary Material.

*R*_0_ for the SIR model was estimated to a mean of 1.88 [95% CI 1.46–2.41]. In models with antibody loss and loss of immunity, *R*_0_ was 1.66 [95% CI 1.35–2.08] (SIRS) and 1.77 [95% CI 1.45–2.22] (SIRS with slow antibody loss). In models with antibody loss and lifelong immunity, *R*_0_ was 2.25 [95% CI 1.67–3.07] (SIRN) and 2.13 [95% CI 1.60–2.78] (SIRN with slow antibody loss) (Figure 4 and supplementary Table S1). The percentage of the population (averaged across the best 1000 simulations) that needs to be vaccinated to eradicate the disease was 47, 56 and 53% for the SIR, SIRN and SIRN with slow antibody loss, respectively. The *N* class represented an averaged 25% (range 4–45%) and 18% (range 4–33%) of the entire female population in the SIRN and SIRN with slow antibody loss, respectively. In the sample used to estimate the age–seroprevalence curve with the ABC-SMC, they represented only 4% (range 0–13%) and 2% (range 0–8%) of the 478 female elk sampled. The waiting time in the recovered class reflects the duration of antibodies at detectable levels. This waiting time was estimated for the SIRN and SIRN with slow antibody loss model from the best 1000 simulations and had a median of 4 years (range 1–30) and 8 years (range 5–24), respectively.

## Discussion

Our results suggest that antibody loss and subsequent lifelong immunity are mechanisms explaining the decline in brucellosis seroprevalence with age observed in elk. The declining seroprevalence among elk aged 10 and older was unlikely to be due to survival differences. We found no effect of seropositive status on elk survival, and even using the upper 95th percentile of the survival difference was not enough to create the observed decline in seroprevalence. To our knowledge, this is the first study of a wildlife species to estimate the probability of antibody loss and account for this phenomenon in the estimation of vaccination coverage.

Most wildlife diseases exhibit a monotonic increase in seroprevalence with age (Cleaveland et al. 2000; Farrington et al. 2001; Packer et al. 1999). Therefore, the (serological positive) recovery stage is assumed to be lifelong and antibody loss is neglected in most systems focusing on seroprevalence. Our results challenge the assumption of lifelong antibodies for brucellosis in elk. First, evidence of antibody loss is supported by our empirical dataset in 25 females. Secondly, the age–seroprevalence curve of brucellosis decreases with age, which our model suggests a consequence of antibody loss and subsequent immunity not involving antibodies. The average probability of antibody loss estimated from the GLM approach (0.07 year^−1^) was smaller than the probability estimated using the ABC-SMC method for the equivalent model with antibody loss and lifelong immunity (0.14 year^−1^), but with an overlapping of the 95% confidence interval and 95% credible interval of these estimates.

The ability to detect antibody loss will depend on the proportion of the population affected by this phenomenon and the sample size available for that part of the population. A small number of antibody loss events that occur one year after infection may be caused by either false positives to the test or by individuals that were exposed to the disease but did not develop a strong antibody response. Furthermore, the large majority of antibody loss events may only appear after several years (i.e., slow antibody loss models), thus affecting a very limited proportion of the adult population. For example, we estimated that only 18% of the entire population belongs to the negative *N* class. Age distributions are generally biased toward a small percentage of old individuals, making it difficult to accurately estimate whether seroprevalence is decreasing or not in old individuals. This is the case in our study, although the ABC-SMC inference proved to be sensitive enough to estimate parameters despite a small sample size. Particular effort in sampling old age categories will likely refine those estimations and perhaps allow discriminating between more complex scenarios (e.g., including disease recrudescence).

The vaccination of juveniles in this population could also affect antibody prevalence in that age category. However, if the vaccination of juveniles resulted in detectable antibodies the following year, one would expect almost 100% seroprevalence among one- to three-year-old elk. Instead, these ages tended to have very low seroprevalence that was gradually increasing as would be expected from natural exposures rather than a juvenile only vaccination program that covers the majority of the juvenile population (Scurlock and Edwards 2010). In addition, a parallel study on elk in the region indicated that 16% of seropositive pregnant female elk had abortions compared to only 2% of seronegative elk (Cross et al. 2015; Etter and Drew 2006). Thus, we believe our results apply to natural exposures of elk to *B. abortus* and the subsequent loss of antibodies to those natural exposures, rather than some artifact of the vaccination program (Maichak et al. 2017).

The percentage of the *N* class in the population should also affect the estimation of other epidemiological parameters including vaccination coverage. Estimations of *R*_0_ increased by less than 20% in models accounting for antibody loss, because this phenomenon affects only a small percentage of the population. *R*_0_ for brucellosis has been estimated to be 1.76 in bison (Hobbs et al. 2015), but no formal calculation has been made for elk. In this study, *R*_0_ was estimated to be 2.13 for the model with slow antibody loss that best fit the data and 1.88 for the model without antibody loss. If antibody loss happens early in life relative to the species lifetime, we expect that the underestimation of a higher percentage of negative individuals wrongly considered as susceptible might affect the estimation of *R*_0_ more considerably. The estimated vaccination coverage needed to eradicate brucellosis in this elk population, directly derived from *R*_0_ estimates, is an essential tool for planning vaccination programs such as those established at Wyoming feedgrounds (Scurlock and Edwards 2010). Current vaccines available for elk are not fully protective against infection (Cross et al. 2007; Kreeger et al. 2002; Olsen et al. 2006), but our results suggest that if an effective vaccine was available, then the vaccination program should plan to cover at least half of the female population to eradicate the disease. This amount of coverage is easily attained on the supplemental feeding grounds where managers routinely remotely vaccinate in excess of 90% of the calves every year, but would be very difficult in other free-ranging elk populations. Further work would be needed to estimate vaccination coverage on a more realistic scenario using a partially protective vaccine (Arenas-Gamboa et al. 2009).

Our model predictions favor the existence of a lifelong adaptive immune response to brucellosis after the first infection, but that this immunity is not reflected in the current serological assays. Although there is an incomplete understanding of the immune mechanism involved in *Brucella* infection, this prediction is in agreement with the proposition that T cell-mediated immunity is an important mechanism to fight brucellosis infection (Yingst and Hoover 2003). In fact, cell-mediated immunity has been suggested both as a mechanism explaining protection to the RB51 vaccine in cattle (Stevens et al. 1994) and as the most important immunological long-term response for bison brucellosis (Olsen 2010). Although cell-mediated immunity can also trigger antibody production during a secondary infection, T cells do not seem to be associated with antibody production in the brucellosis immunological response (Gill et al. 1992). Given the low number of samples in older individuals used to estimate the age–seroprevalence curve, a more conclusive discrimination between titer loss models with or without lifelong adaptive immunity (SIRN versus SIRS) will require further work. This could include the noninvasive measurement of specific cell-mediated immunity proteins against brucellosis such as cytokines in individuals experiencing antibody loss. Immunological scenarios combining both a humoral and cell-mediated long-term immunity are plausible, but our empirical data do not have enough statistical power to discriminate between more complex scenarios that could all generate a decrease in seroprevalence with age. Likewise, further work is needed to establish the importance of disease latency and recrudescence of brucellosis in elk, and whether that could influence the observed patterns of seropositivity.

Other than antibody loss, disease-induced mortality can also generate a decrease in prevalence in older individuals (Benavides et al. 2012). A study on bison showed that brucellosis alone had little or no effect on survival (Joly and Messier 2005). Likewise, our analyses show no evidence for brucellosis-induced mortality in female elk, although parameter estimations were highly variable. Furthermore, we did not find a decrease in seroprevalence with age when accounting for disease-induced mortality using the formula described by Heisey et al. (2006). In other systems, antibody loss and disease-induced mortality may co-occur and it may be difficult to disentangle these two mechanisms using only the relationship between age and seroprevalence. Ancillary data on one of the factors in isolation may be required. Alternatively to disease-induced mortality, reduced contact between old individuals and other members of the population could prevent them from becoming re-exposed to brucellosis, also generating lower seroprevalence in that age category. However, previous studies have found inconclusive evidence of individual differences in elk–fetus contact rates at feedgrounds (Creech et al. 2012) or low individual variation in contact between females compared to other factors (Cross et al. 2013).

## Conclusion

Our study suggests antibody loss and subsequent cell-mediated lifelong immunity as the within-host dynamics of brucellosis in elk. Antibody loss has also been suggested in older bison individuals (Joly and Messier 2004). Therefore, it may be a common issue that is seldom recognized in wildlife disease studies. Our results also suggest that antibody loss and subsequent loss of immunity only generate a minor decrease in seroprevalence with age, which could be undetected in small sample sizes and confounded with no antibody loss. This calls for a re-evaluation of the assumption of long-lived antibodies made in most studies estimating epidemiological parameters from serological data.

## Electronic supplementary material

Below is the link to the electronic supplementary material.

Supplementary material 1 (DOCX 516 kb)

Supplementary material 2 (XLSX 35 kb)
