# Supplementary material for: Estimating Loss of Brucella Abortus Antibodies from Age-Specific Serological Data In Elk
Source: Ecohealth. 2017 May 15;14(2):234–43. doi: 10.1007/s10393-017-1235-z (PMC5486471; doi:10.1007/s10393-017-1235-z)
Supplement: Supplementary file 1 — Supplementary material 1 (DOCX 516 kb) [file 10393_2017_1235_MOESM1_ESM.docx]

Ecohealth

**Estimating loss of *Brucella abortus* antibodies from age-specific serological data in elk**

**Authors: J.A. BENAVIDES, D. CAILLAUD, B.M. SCURLOCK, E.J. MAICHAK, W.H. EDWARDS, P.C. CROSS**

Supplementary Material

**Section 1.** **Blood sample collection and serological methods**

For both datasets, blood sample collection and serological methods used in this study have been described elsewhere ([Cross et al. 2007](#_ENREF_6), [Cross et al. 2010](#_ENREF_5), [Scurlock and Edwards 2010](#_ENREF_22)), and are briefly summarized here. Serological assays were conducted and interpreted using current National Veterinary Services Laboratories protocols for the card test, plate agglutination, rivanol precipitation–plate agglutination, and complement fixation. A competitive ELISA (cELISA) was used to discriminate field strain from vaccine strain (*i.e.* strain 19) titers (n = 29), which were excluded from this study ([Van Houten et al. 2003](#_ENREF_27)). Reactors were those animals with positive card tests, rivanol ≥1:25 or higher, CF of 2+ at 1:20, and SPT ≥1:100 or higher. Serological profiles were categorized using the United States Department of Agriculture's brucellosis eradication uniform methods and rules for cervids (APHIS 91-45-013). Less than 1% of the serological tests were categorized as suspect, which we included as positive test results. All data is available on Supplementary Material Tables. The first dataset used to discriminate between different models of brucellosis consisted of 371 individual females with known ages (1.5 years and older) collected from 1993 to 2012. Eighty-two (22%) of individuals were sampled multiple times in different years (median: 2, range: 2 - 7 times) and remained within the analysis to increase the number of individuals at older ages. Choosing one randomly selected sample per individual did not change the results. By focusing on females, we utilize the population segment most relevant to transmission of *B. abortus* ([Cheville et al. 1998](#_ENREF_4))*.* Age was estimated in most females (n= 377) using tooth eruption patterns for individuals caught as calves or yearlings that were ear tagged and subsequently caught as adults. The cemmentum annuli method was used for the remaining individuals ([Keiss 1969](#_ENREF_17)) (n = 13).

**Section 2. Estimation of antibody loss using a Generalized Linear Model**

In this analysis that uses the second dataset from females sampled multiple times, we used the first positive sample available per female as a starting point and samples for each individual were included for subsequent years until encountering the first negative sample or until no more positive samples were available. We then analyzed the data by fitting a GLM binomial model to antibody loss as the dependent variable, with a complementary log-log link function, and using log(X) as an offset term, where X equals the number of years after the first positive test. This allowed calculating a minimal per-year probability of antibody loss from a positive to a negative status, which is expected to be $1-e^{-e^{Bo}}$, where *Bo* was the intercept of that regression. The presence of false positive could bias the results of this analysis. In particular, false positives that test negative for the subsequent sampling year could be mistakenly interpreted as an antibody loss event, increasing the antibody loss rate estimated by the GLM analysis. Thus, we estimated an approximate percentage of false positives in our population to determine the impact of this phenomenon in our analyses. On average, combining the serological tests used in this study should lead to a test specificity higher than 90% for elk ([Gall et al. 2001](#_ENREF_14), [Schumaker et al. 2010](#_ENREF_21)). The expected percentage of false positives FP can thus be estimated as $\mathrm{FP}=\left( 1-prevalence \right)\times\left( 1-specificity \right)\times100.$

**Section 3. Simulation models of brucellosis dynamics**

The three following models were simulated and compared using the ABC method:

***‘No antibody loss’ model:*** In this S-I-R model, each individual can move through three different classes: susceptible, infectious and recovered ([Keeling and Rohani 2008](#_ENREF_16)). Susceptible individuals can be infected with probability *p_t_* such that $p_{t}= 1-\left( 1-\beta\right)^{I(t)}$, where $\beta$ is the transmission probability from a single infected individual to a susceptible individual. The year time step allows coverage of the entire infectious period of brucellosis during the abortion period from February to June. Each infected individual passes from infectious to recovered with probability *γ.* This model assumes individuals exposed to the disease test seropositive throughout their lifespan, *i.e.* no antibody loss ([Dobson and Meagher 1996](#_ENREF_9), [Xie and Horan 2009](#_ENREF_29)). Brucellosis can relapse from a recovery stage to an infectious stage in humans ([Pellicer et al. 1988](#_ENREF_19)) but this has not been described in elk. Therefore, we did not include a relapse stage in our model. Given the discrete nature of the model, individuals stayed at least one year in each disease class, except for the infectious state, where individuals could recover and move to the *R* class the same year they became infectious. This was demonstrated by a controlled study on brucellosis dynamics in female elk ([Thorne et al. 1978](#_ENREF_25)), where seven out of 13 naturally infected females aborted the same year they were infected.

***‘Antibody loss and loss of immunity’ model****:* This S-I-R-S model has the same structure and probabilities as the above SIR model but it also includes a probability *θ* that an individual will transit from the *R* class back to *S*. This novel model reflects a scenario with antibody loss but no lifelong immunity: the individual can become susceptible to the disease again. A slightly different version of this model was also implemented, which included a multi-compartmental ‘box-car’ approach ([Keeling and Rohani](#_ENREF_16)) to create recovered periods (class *R*) that were roughly log-normally distributed ([Lloyd 2001](#_ENREF_18), [Wearing et al. 2005](#_ENREF_28)). This model was referred to as the ‘Slow antibody loss and loss of immunity model’. The number of box cars was fixed at five after comparing it with a three and four box-cars (see results). In this model, the *R* class is divided into five stages *(R_1_* to *R_5_*) with probably *θ* of transiting between stages and to pass from *R_5_* to the *S* class. This model illustrates a scenario where the permanence time in the R class is not necessarily described by an exponential distribution.

***‘Antibody loss and lifelong immunity’ model:***ThisS-I-R-N model has the same structure as the SIR model but also includes a Negative (*N*) class and a probability *δ* of transiting from the *R* to the *N* class. In this version, antibody loss is followed by lifelong immunity for individuals in the *N* class, conferred, for example, by T-cell mediated immunity ([Yingst and Hoover 2003](#_ENREF_30)). Therefore, negative individuals stay negative until they die. A five box-car version of this model, referred to as the ‘Slow antibody loss and lifelong immunity’ model, was also implemented.

In all three models, we assume that individuals in the *I* and *R* classes test positive for antibodies whereas individuals in the *S* and *N* classes test negative. Simulations were initiated by randomly introducing one infectious individual into a population of 800 susceptible elk, which correspond to the averaged empirical size (*i.e.* 792 animals) of the Grey’s River feedground. In all models, age increased with each annual time step. The model is stochastic in that binomial trials governed the random outcomes of disease transmission and mortality. Following disease transmission, mortality and reproduction of the 800 individuals (similar to the averaged population in the feedground) took place as a single death/birth pulse at the end of the year. Each individual died and was replaced by a new susceptible newborn with probability *µ_i_*, where *i* was the age of the individual (1 to 26). Mortality rates were set to µ = 0.3 for age 1 ([Singer et al. 1997](#_ENREF_23)), 0.1 for ages 2-18 ([Brodie et al. 2013](#_ENREF_3)), and 0.5 for 19+ to account for senescence and higher predation risk in older animals ([Eberhardt et al. 2007](#_ENREF_10)). Simulations were run until the model reached steady-state equilibrium, *i.e.* all classes *S*, *I*, *R* and *N* did not change with time. We coded and ran all simulations using Delphi v6 computing software (2006, Borland, Inc.).

***Approximate Bayesian Computation - Sequential Monte Carlo (ABC-SMC)***

The ABC-SMC algorithm simulates a large number of datasets using parameter values drawn in prior distributions. Each simulated dataset is compared to the observed data and a “distance” metric is calculated that quantifies how similar the simulation is to the observation. We used the sum of squared differences between the observed and simulated prevalence at each age category weighted by the sample size, given by $SS= \sum_{i=1}^{19} {(e_{i}{-o}_{i})}^{2}*A_{i}$, where $A_{i}$ is the number of samples collected at age *i*. For this comparison, we use a random sampling from the simulated population of 800 individuals in the last 20 years, equivalent to the empirical number of samples per age category (*i.e*. same number of individuals per age category). A filtering algorithm (SMC) is then applied, that allows discarding parameter vectors that lead to “bad”, distant simulations, and generates a large number of parameter vectors that lead to “good” simulations. The selected parameter values form the posterior distributions of the model. The SMC algorithm proceeds in several steps. Each step consists of generating a large number of simulations and applying the filtering algorithm to discard the simulations least resembling the observed data. The selected parameter vectors are then perturbed and used to generate the simulation of the following step. Step after step, the generated simulations become more and more similar to the data. In the first step of the algorithm 50 000 particles, each “particle” corresponding to the set of model parameters, are randomly chosen from the prior distribution. Then, simulations from our stochastic epidemiological model are performed for each particle, the summarized metric calculated for each simulation, and then 1000 closest simulations to the empirical data retained, referred to as a population of particles. Then, a uniform density kernel around the population of particles selected is fitted and 50 000 particles are resampled according to the weight of each selected particle in the population. Weights are calculated using the prior distribution and the weights of particles from the previous population (equal to one for the first particle population), as described in Toni et al. (2009). These steps are then repeated several times to generate a series of populations of particles that should evolve to the target posterior ([Beaumont 2010](#_ENREF_1)). The SMC algorithm described here is slightly modified from Toni et al.’s original formulation, but is mathematically equivalent. In the original formulation, each parameter vector is accepted or rejected immediately after the corresponding dataset has been generated. In our case, the selection procedure is applied only every 50 000 simulations. Each ABC-SMC procedure was run for a total of seven populations, previously tested to converge to stable posterior distributions.

***Evaluation of the ABC approximation***

In order to evaluate the acceptance method used in the ABC, we compared four approaches: the ABC using 10 000, 50 000 or 100 0000 particles per population, and an approach using a tolerance sequence method as described in Toni et al. 2009 with 10 000 simulations. In the latter, the sequence tolerances were set as ε_1_ = 50, ε_2_ = 25, ε_3_ = 12.5, ε_4_ = 10, ε_5_ = 8.75, ε_6_ = 7.5, ε_7_ = 6.25, where simulations will only be retained if the *SS* metric is smaller than the established tolerance threshold for particle populations 1 to 7. Posterior distributions for the four methods are given in the following figure:

**Figure S1:** Posterior distributions using four different approaches. Each line represents the posterior distribution of each parameter using 10 000 particles (yellow), 50 0000 particles (orange), 100 000 particles (red) and a tolerance sequence (black). Vertical lines illustrate the mode value for each estimation.

***
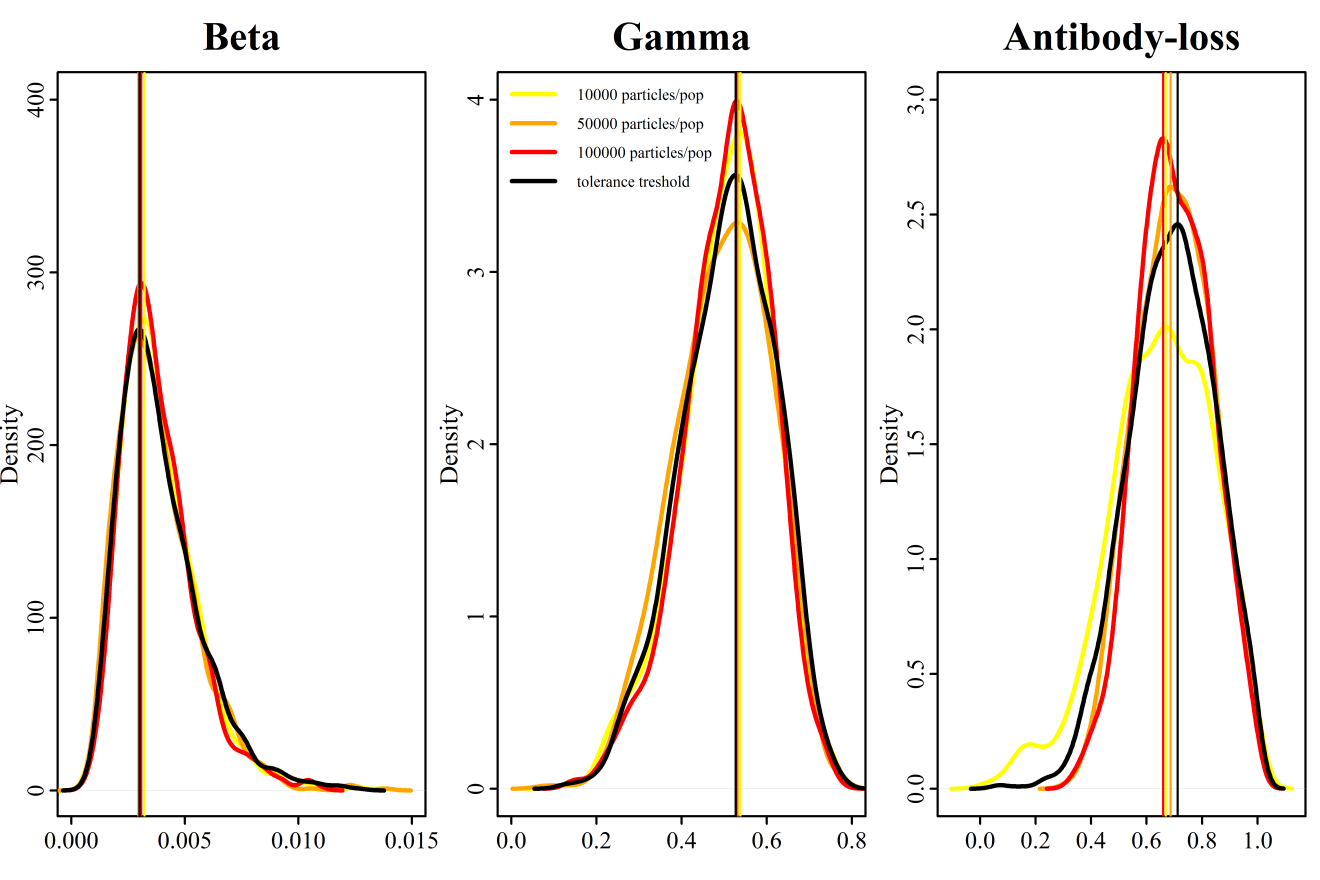
***

Only minor differences in the estimation of the three parameters are observed. The main difference is a reduction in the confidence interval of the antibody loss parameter when using the 50 000 and 100 000 particles, compared to the other two methods. No significant differences were detected between the ABC approach using 50 000 and 100 000 particles. Therefore, we used the ABC method with 50 000 particles per population in all models.

***Prior assumptions***

Uniform uninformative prior distributions ranging from 0 to 1 are applied to all individual epidemiological parameter probabilities (*β*, *δ*, *θ*), except for the probability *γ*. An informative prior was used for *γ*, estimated from a previous elk infection trial ([Thorne et al. 1978](#_ENREF_25)). For this prior, we use a $Beta\left( k;l \right)$ distribution ([Fink 1997](#_ENREF_12), [Bousquet 2008](#_ENREF_2)). Parameters k and *l* were estimated from Thorne’s study as: *k* = number of females not aborting after the first year - 1 and *l* = number of females aborting after the first year-1, with *k* = 5 and *l* = 6. Choosing a strong prior for γ allows a better estimation of parameter *β*, since both are negatively correlated in SIR models. Using a flat prior on γ result in minor differences on parameter estimations of the model that fitted the data the best (Figure S2).

**Figure S2.** Posterior distributions for the SIRN ‘Slow antibody loss and lifelong immunity’ model using a uniform or informative prior

**
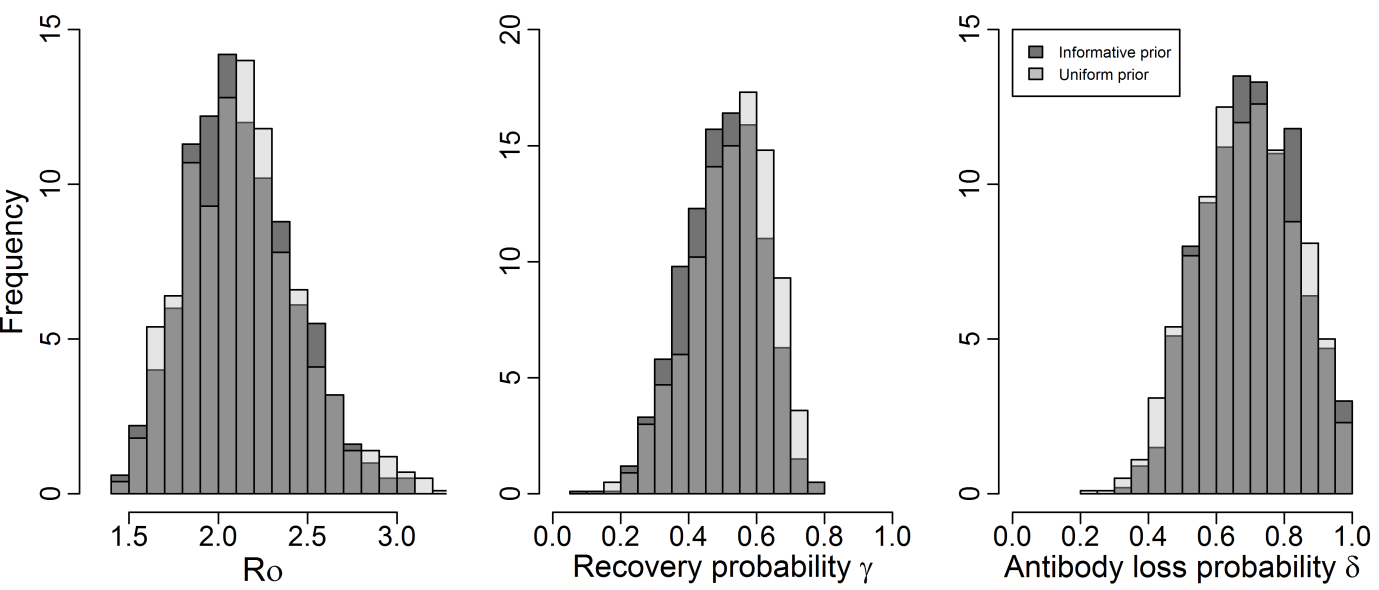
**

***Parameter identifiability***

In order to test whether our ABC-SMC approach was able to accurately estimate posterior distributions from different model scenarios given our empirical dataset, we simulated age-seroprevalence data from our five models (SIR, SIRN, SIRN-box, SIRS, SIRS-box). We used the mode of the estimated distribution for each scenario presented in Table S1 as parameter values for each scenario. We simulated 100 age-seroprevalence curves from our simulation model and run the ABC-SMC on each of those curves to re-estimate posterior distributions. We then observed whether these posterior distributions were able to estimate the value of the parameter used to simulate the age-seroprevalence curves. In all scenarios, the ABC-SMC was able to estimate posterior distributions with a mode close to the simulated value (Figure S3). The SIRS and SIRS-box model showed the highest variation in posterior distribution of the antibody loss probability, although the mode of the distribution remained close to the simulated value.

**Figure S3.** Estimated parameter distributions for 100 simulations of the SIRN, SIRN-box, SIRS, SIRS-box and SIR models (from top to bottom). Vertical lines show the parameter value use to simulate the data, which corresponds to the mode of the posterior distribution estimated in our study (Table S1). Uniform uninformative prior distributions ranging from 0 to 1 were used for probabilities *β* and *θ*, whereas *γ* was taken from a $Beta\left( k;l \right)$ distribution, with *k* = 5 and *l* = 6 (see above section for details on this prior). Given computational limitations, the ABC-SMC procedure was run for 4 populations.


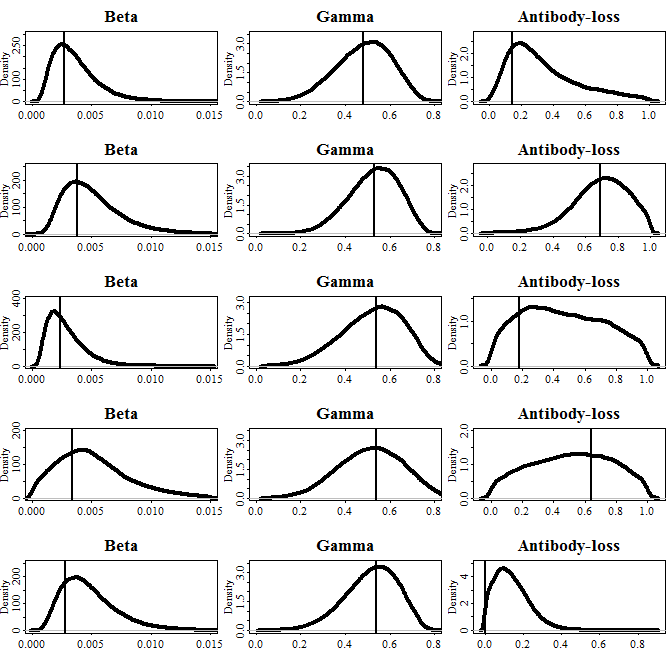


***Model comparison***

The ABC approach does not require a likelihood-function for parameter selection, which is difficult to calculate for complex simulation models such as the one presented here. Model selection in ABC-SMC has been traditionally performed by calculating a Bayesian factor when running the ABC procedure for different competitive models simultaneously ([Toni et al. 2009](#_ENREF_26), [Csillery et al. 2010](#_ENREF_7)). However, the use of the Bayes factor for discriminating between different models has been criticized, particularly when the number of summary statistics is low as in this study ([Didelot et al. 2011](#_ENREF_8), [François and Laval 2011](#_ENREF_13), [Robert et al. 2011](#_ENREF_20)). Therefore, model discrimination should be performed with caution. We discriminate between models based on their ability to reproduce the empirical decline in seroprevalence observed in adults and on their goodness of fit based on the summary statistics itself. Furthermore, the posterior distribution of the antibody loss parameter in the SIRN-box model supports that the antibody loss parameter is not close to zero, which favors a SIRN model over a SIR model. This discrimination problem will probably be solved in the near future with the development of new approaches for model comparison ([Didelot et al. 2011](#_ENREF_8), [François and Laval 2011](#_ENREF_13))] and the improvement of summary statistics choices ([Fearnhead and Prangle 2012](#_ENREF_11)), which may become available for the ABC-SMC approach.

**Section 4. Ro calculation from simulated models using ABC posteriors**

An approximation of *R*_0_ was calculated based on the parameters of the discrete models (SIR, SIRS and SIRN) estimated using the ABC. In a continuous model, *R*_0_, is the expected transmission rate of a single infected individual multiplied by the expected time in the infectious class. In our discrete model, the expected duration, $\tau$, in the *I* class (in years) is the mean of the geometric distribution, whereby $\tau=\frac{\left( 1-f \right)}{f}$, with $f=$ probability of leaving the *I* class. We assumed the probability of dying was the average of the age-specific mortality probabilities weighted by the age distribution ($\bar{\mu}$ = 0.13). As a result, $f$ = 1 – P(staying in *I*) *=* 1 – P(not recovering) x P(staying alive) = $1-\left( 1-\gamma\right) \times(1-\bar{\mu)}$ $=\gamma+\bar{\mu}\left( 1-\gamma\right)$. The probability that any given individual is infected by a single infectious individual per year is 1-(1-*β*)^1^, or just *β*. So, in our model the expected number of infections per year for a single infectious individual is *β*Z at the start of the epidemic, where Z is the total population size. This assumes that the effect of a depleting susceptible pool from the contamination of a single infectious individual at the start of the epidemic, resulting in the transition of a few individuals from S to I, is negligible. Note that although we assumed the transmission rate was a function of the number of infected (*p*_t_ = 1-(1-* β*)^It^), our population was assumed to be constant in the model. As a result, changes to our population size Z would have resulted in a concomitant change in the estimated *β* when estimating parameter values using the ABC-SMC approach from the observed seroprevalence. We calculated $R_{o}$ as:

$$R_{0}\approx\beta\times Z\times\frac{\left( 1-f \right)}{f}\approx\beta\times Z\times\frac{\left( 1-(\gamma+\left( 1-\gamma\right)\times\bar{\mu}) \right)}{(\gamma+\left( 1-\gamma\right)\times\bar{\mu})}$$

This is an approximation due to the discrete time approximation that allowed us to more easily compare the results to our annual data, but where the order of operations in the model may have a minor effect on some of the estimates.

**Section 5. Analysis of survival based on serological status**

We tested whether elk survival was affected by serological status by using collar data of 258 female elk. In a first analysis, survival was assessed as a binomial response of whether an animal survived or not by December 1^st^, which represents the end of most hunting seasons. This analysis included 328 elk-year events where elk either died during the year or were known to be alive at the end of hunting season. The impact of serological status was tested using a GLM model with binomial response including both serological status and feedground location as explanatory variables. A second analysis was performed using the Cox-proportional hazard model with the Survival package in R. This analysis included the time of death for the 53 death events or the day when the collar was dropped (censoring time) using day of year, rather than calendar date as the baseline. Data from individuals collared for several years were considered as independent survival events for each year, but similar results were found when analyzing data using a mixed-effect model with the coxme package in R.

***Predicting the age-seroprevalence curve with disease induced mortality.***

Following Heisey *et al.* (2006), we estimated the expected seroprevalence at each age class accounting only for disease-induced mortality and no antibody loss as:

$$P_{i}=\frac{1-e^{-\left[ \left( \lambda-d \right)\times i \right]}}{1-\frac{d}{\lambda} \times e^{-\left[ \left( \lambda-d \right)\times i \right]}}$$

Where *i* = age, $d=$ disease-induced mortality from the baseline mortality and λ = the force of infection ([Heisey et al. 2006](#_ENREF_15)). From the above analysis, $\lambda\approx0.04$ and the maximal estimate of disease-induced mortality *M* from the GLM analysis is $M\approx2.52 \times0.13 \approx0.33$. Therefore,$d\approx0.33-0.13 \approx0.2$ and the estimated age-seroprevalence curve is:


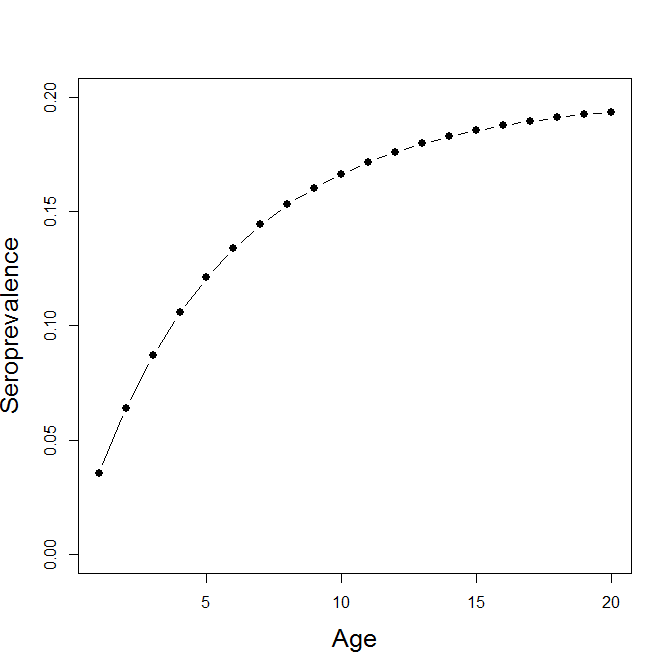


Supplementary Table S1: Posterior distributions summaries for each model calibrated by the ABC-SMC

| **Parameters** | **SIR** | **SIRS** | **SIRS-box** | **SIRN** | **SIRN-box** |
| --- | --- | --- | --- | --- | --- |
| *β* (*S* to *I* class) | 2.8 :3.01±1.30*  (1.00, 8.05)** | 2.33: 3.33±1.79* (0.9, 7.8) | 2.64:3.34±1.40*  (1.29, 6.60) | 2.74: 3.90±1.92*  (1.37, 8.78) | 2.95: 3.78±1.73* (1.41, 7.84) |
| *γ* (*I* to *R*) | 0.54: 0.48±0.11 (0.25, 0.66) | 0.54: 0.52±0.14 (0.20, 0.75) | 0.54 :0.52±0.11 (0.28, 0.70) | 0.48: 0.49±0.12 (0.25, 0.70) | 0.53: 0.50±0.11 (0.27, 0.69) |
| *δ* (*R* to *N*) | **-** | - | - | 0.14: 0.15±0.08 (0.04, 0.32) | 0.69: 0.70±0.14 (0.45, 0.96) |
| *θ* (*R* to *S*) | - | 0.18: 0.26±0.17 (0.04, 0.75) | 0.72:0.64±0.19  (0.25, 0.97) | - | - |
| *R_0_* | 1.84: 1.88±0.24 (1.46, 2.41) | 1.58: 1.66±0.20 (1.35, 2,08) | 1.71: 1.77±0.20 (1.45, 2.22) | 2.17: 2.25±0.35 (1.67, 3.07) | 2.05: 2.13±0.30 (1.60, 2.78) |

* All values need to be multiplied by 10^-3^

**Values shown are as follows: mode: mean+- SD (95% confidence interval)

**REFERENCES**

Beaumont, M. A. (2010). "Approximate Bayesian computation in evolution and ecology." Annual Review of Ecology, Evolution, and Systematics **41**: 379-406.

Bousquet, N. (2008). "Diagnostics of prior-data agreement in applied Bayesian analysis." Journal of Applied Statistics **35**(9): 1011-1029.

Brodie, J., H. Johnson, M. Mitchell, P. Zager, K. Proffitt, M. Hebblewhite, M. Kauffman, B. Johnson, J. Bissonette and C. Bishop (2013). "Relative influence of human harvest, carnivores, and weather on adult female elk survival across western North America." Journal of Applied Ecology **50**(2): 295-305.

Cheville, N. F., D. R. McCullough and L. R. Paulson (1998). Brucellosis in the greater Yellowstone area, National Academies Press.

Cross, P. C., E. K. Cole, A. P. Dobson, W. H. Edwards, K. L. Hamlin, G. Luikart, A. D. Middleton, B. M. Scurlock and P. J. White (2010). "Probable causes of increasing brucellosis in free-ranging elk of the Greater Yellowstone Ecosystem." Ecological Applications **20**(1): 278-288.

Cross, P. C., W. H. Edwards, B. M. Scurlock, E. J. Maichak and J. D. Rogerson (2007). "Effects of management and climate on elk brucellosis in the Greater Yellowstone Ecosystem." Ecological Applications **17**(4): 957-964.

Csillery, K., M. G. B. Blum, O. E. Gaggiotti and O. Francois (2010). "Approximate Bayesian computation (ABC) in practice." Trends in ecology & evolution **25**(7): 410-418.

Didelot, X., R. G. Everitt, A. M. Johansen and D. J. Lawson (2011). "Likelihood-free estimation of model evidence." Bayesian analysis **6**(1): 49-76.

Dobson, A. and M. Meagher (1996). "The population dynamics of brucellosis in the Yellowstone National Park." Ecology: 1026-1036.

Eberhardt, L., P. White, R. A. GARROTT and D. Houston (2007). "A Seventy‐Year History of Trends in Yellowstone's Northern Elk Herd." The Journal of wildlife management **71**(2): 594-602.

Fearnhead, P. and D. Prangle (2012). "Constructing summary statistics for approximate Bayesian computation: semi-automatic approximate Bayesian computation." Journal of the Royal Statistical Society: Series B (Statistical Methodology) **74**(3): 419-474.

Fink, D. (1997). "A compendium of conjugate priors." See <http://www>. people. cornell. edu/pages/df36/CONJINTRnew% 20TEX. pdf: 46.

François, O. and G. Laval (2011). "Deviance information criteria for model selection in approximate bayesian computation." Statistical Applications in Genetics and Molecular Biology **10**(1).

Gall, D., K. Nielsen, L. Forbes, W. Cook, D. Leclair, S. Balsevicius, L. Kelly, P. Smith and M. Mallory (2001). "Evaluation of the fluorescence polarization assay and comparison to other serological assays for detection of brucellosis in cervids." Journal of wildlife diseases **37**(1): 110-118.

Heisey, D. M., D. O. Joly and F. Messier (2006). "The fitting of general force-of-infection models to wildlife disease prevalence data." Ecology **87**(9): 2356-2365.

Keeling, M. J. and P. Rohani (2008). Modeling infectious diseases in humans and animals, Princeton University Press.

Keiss, R. E. (1969). "Comparison of eruption-wear patterns and cementum annuli as age criteria in elk." The Journal of Wildlife Management: 175-180.

Lloyd, A. L. (2001). "Realistic distributions of infectious periods in epidemic models: changing patterns of persistence and dynamics." Theoretical Population Biology **60**(1): 59-71.

Pellicer, T., J. Ariza, A. Foz, R. Pallares and F. Gudiol (1988). "Specific antibodies detected during relapse of human brucellosis." Journal of Infectious Diseases **157**(5): 918-924.

Robert, C. P., J.-M. Cornuet, J.-M. Marin and N. S. Pillai (2011). "Lack of confidence in approximate Bayesian computation model choice." Proceedings of the National Academy of Sciences **108**(37): 15112-15117.

Schumaker, B. A., J. A. K. Mazet, B. J. Gonzales, P. H. Elzer, S. K. Hietala and M. H. Ziccardi (2010). "Evaluation of the Western immunoblot as a detection method for Brucella abortus exposure in elk." Journal of wildlife diseases **46**(1): 87-94.

Scurlock, B. M. and W. H. Edwards (2010). "Status of brucellosis in free-ranging elk and bison in Wyoming." Journal of Wildlife Diseases **46**(2): 442-449.

Singer, F. J., A. Harting, K. K. Symonds and M. B. Coughenour (1997). "Density dependence, compensation, and environmental effects on elk calf mortality in Yellowstone National Park." The Journal of wildlife management: 12-25.

Sjogren, M. H., H. Tanno, O. Fay, S. Sileoni, B. D. Cohen, D. S. Burke and R. J. Feighny (1987). "Hepatitis A virus in stool during clinical relapse." Annals of internal medicine **106**(2): 221-226.

Thorne, E. T., J. K. Morton, F. M. Blunt and H. A. Dawson (1978). "Brucellosis in elk II. Clinical effects and means of transmission as determined through artificial infections." Journal of Wildlife Diseases **14**(3): 279.

Toni, T., D. Welch, N. Strelkowa, A. Ipsen and M. P. H. Stumpf (2009). "Approximate Bayesian computation scheme for parameter inference and model selection in dynamical systems." Journal of the Royal Society Interface **6**(31): 187-202.

Van Houten, C. K., E. L. Belden, T. J. Kreeger, E. S. Williams, W. H. Edwards, E. T. Thorne, W. E. Cook and K. W. Mills (2003). "Validation of a Brucella abortus competitive enzyme-linked immunosorbent assay for use in Rocky Mountain elk (Cervus elaphus nelsoni)." Journal of wildlife diseases **39**(2): 316-322.

Wearing, H. J., P. Rohani and M. J. Keeling (2005). "Appropriate models for the management of infectious diseases." PLoS Medicine **2**(7): e174.

Xie, F. and R. D. Horan (2009). "Disease and behavioral dynamics for brucellosis control in elk and cattle in the Greater Yellowstone Area." Journal of Agricultural and Resource Economics: 11-33.

Yingst, S. and D. L. Hoover (2003). "T cell immunity to brucellosis." Critical reviews in microbiology **29**(4): 313-331.
